# Exploring haematopoietic stem cell dynamics through mitochondrial mutation profiling

**DOI:** 10.1186/s43556-024-00190-2

**Published:** 2024-07-10

**Authors:** Yongming Xia, Ruixiu Chen, Shiwei Duan

**Affiliations:** 1 Department of Hematology, Yuyao People’s Hospital of Zhejiang Province, The Affiliated Yangming Hospital of Ningbo University, Yuyao, 315400 Zhejiang China; 2 Department of Clinical Medicine, Hangzhou City University, Hangzhou, Zhejiang China

**Keywords:** Haematopoietic stem cells, Mitochondrial DNA mutations, Clonal dynamics, Single-cell sequencing, Lineage reconstruction, Blood disorders

## Main text

In a recent study published in Nature, Weng C et al. developed ReDeeM technology, leveraging natural barcodes—mitochondrial DNA (mtDNA) mutations. This innovative Deep Mitochondrial Mutation Profiling method combines single-cell transcriptomics and chromatin accessibility analysis to accurately capture the natural barcodes of single cells. By significantly improving the mutation detection rate, ReDeeM technology successfully generated clone-resolved single-cell transcriptome and chromatin maps of approximately 150,000 human hematopoietic cells. This offers a new perspective for deeply understanding the clonal behavior of hematopoietic stem cells (HSCs) in maintaining blood system function and in aging and disease [1]. Increasing evidence suggests that HSCs exhibit diverse clonal behaviors in aging and disease, such as clonal hematopoiesis, which is associated with acute myelocytic leukemia (AML) and an increased risk of cardiovascular disease [2]. Consequently, there is a pressing need for comprehensive investigations into HSCs. In comparison, existing labeling methods exhibit disparities and shortcomings. While gene labeling for human HSCs is typically constrained, the use of mtDNA mutations as natural cell barcodes is promising yet complicated by susceptibility to mutations stemming from internal and external stressors, as well as its dual role in diseases [3, 4]. For example, research by Ohta S et al. demonstrated that pathogenic mtDNA mutations may promote tumor formation [3]. Conversely, Gorelick AN et al. found that certain mutations, including Truncating and variants of uncertain significance, may reduce the risk of colorectal cancer and exhibit a protective effect [4]. Consequently, researchers urgently seek more sophisticated, efficient, and precise methodologies to elucidate the cellular dynamics and lineage of HSCs, and their implications in cancer, aging, and related ailments.

Conventional mixed-cell sequencing techniques, such as Bulk sequencing, mask high-frequency mutations in single cells by averaging them, thus impeding the detection of mtDNA mutation frequency and spectrum in cells. To overcome the limitations of existing technologies and further explore the complexity of HSCs, Weng C et al. introduced the ReDeeM technology. Through optimized mtDNA coverage and the application of unique molecular identifiers, ReDeeM technology can detect rare mutations with minimal heterogeneity, reconstruct the lineage tree and cell status of the human hematopoietic system, and offer new insights into human HSC clones [1].

**Fig. 1:**
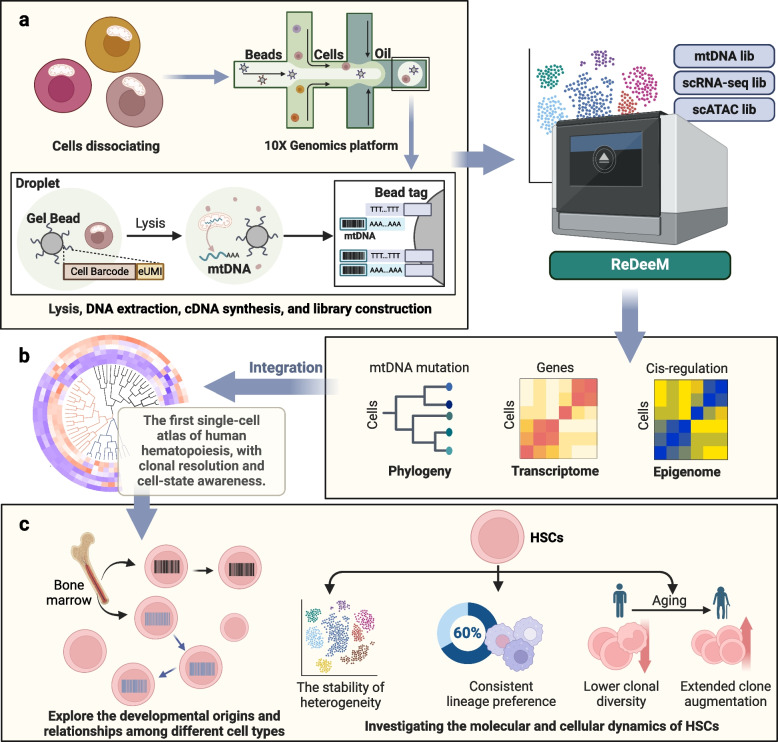
Deciphering Hematopoietic Stem Cell Dynamics through ReDeeM Technology. **a** Schematic diagram illustrating the ReDeeM workflow. First, whole cells containing mitochondrial DNA (mtDNA) are encapsulated in the 10X Genomics platform. The cells are then subjected to lysis, DNA extraction, cDNA synthesis, and library construction. Finally, ReDeeM technology is used to analyze three sequencing libraries—mtDNA, ATAC-seq, and RNA-seq—to accurately capture single-cell barcodes. **b** Utilizing ReDeeM technology, alongside comprehensive transcriptome and epigenome data, and leveraging the molecular status of mtDNA mutations, researchers delineated the first human hematopoiesis map. This map provides unprecedented clonal resolution and cell status information at the single-cell level. **c** Lineage trees generated by ReDeeM technology reveal the origins and relationships of blood and immune cells. The analysis showed that 60% of HSC clones had consistent lineage preferences, while 40% had no clear preference. As age increases, HSC clonal diversity decreases, and the number of multiple expanded clones rises significantly. ReDeeM, single-cell Regulatory Multiomics with Deep Mitochondrial Mutation Profiling; mtDNA, mitochondrial DNA; HSCs, haematopoietic stem cells

Specifically, this technology addresses the resolution constraints of prior cell barcoding and lineage tracing methods. By adapting the droplet-based single-cell multi-component 10X Genomics platform to analyze whole cells, ReDeeM concurrently evaluates three sequencing libraries: mtDNA, ATAC-seq, and RNA-seq. Utilizing endogenous unique molecular identifiers (eUMI) for calibration, wherein cellular barcodes and mtDNA fragment positions serve as eUMIs, significantly enhances mutation detection sensitivity and accuracy (Fig. 1a). Moreover, by deeply detecting naturally occurring mitochondrial DNA mutations, the system provides simultaneous insights into transcriptional status and chromatin accessibility. Applying ReDeeM, researchers scrutinized human CD34^+^ hematopoietic stem and progenitor cells (HSPCs) from healthy donors, uncovering 4,831 high-confidence mtDNA mutations, a tenfold increase over previous methods. Furthermore, they successfully extended this technology to mouse lung adenocarcinoma lineage tracing, corroborating results obtained via the CRISPR method and demonstrating robust reliability at the cellular and clonal levels.

Weng C et al. utilized ReDeeM technology to identify 17 major hematopoietic cell types based on comprehensive transcriptome and epigenome data, revealing that HSCs possess a lower mtDNA mutation burden compared to more differentiated cells. This suggests additional subclonal mtDNA mutations during HSC differentiation. Leveraging these insights, they delineated the first human hematopoiesis map (Fig. 1b) offering clonal resolution and single-cell status information, shedding new light on hematopoietic regulatory mechanisms. By overlaying cell type annotations onto lineage trees derived from multi-omics data, researchers explored the developmental origins and relationships of various blood and immune cell types, revealing extensive subclonal structures.

Further analysis unveiled stable molecular and behavioral heterogeneity among HSCs, evidenced by consistent identification of 14 subpopulations across different time points and donors. These subpopulations exhibited distinct gene expression and transcription factor accessibility, enriching our understanding of HSC function and regulation. Additionally, a correlation between HSC clonal structure, clonal output, and cell type preference emerged, with 60% of HSC clones displaying consistent lineage preference, while 40% lacked discernible preference. These findings offer valuable insights into cell development and state transitions during human hematopoiesis. Notably, analysis of blood samples from elderly donors revealed decreased HSC clone diversity with age, alongside the emergence of multiple expanded clones (Fig. 1c), laying a foundation for deeper investigations into hematopoietic processes in health and disease.

However, the technology is not without limitations. Inconsistencies in experimental results raise doubts about the robustness of conclusions, necessitating further validation. For instance, assessing the accuracy of phylogenetic trees generated by ReDeeM, though supported by the CRISPR method, lacks sufficient experimental breadth, limiting the generalizability of conclusions. Moreover, distinguishing cells lacking mtDNA mutations from those with undetectable mutations remains a challenge in complex human genomes. While the neighbor-joining algorithm demonstrates promise in reconstructing hematopoietic cell phylogenetic trees, its rigorousness warrants scrutiny. These challenges underscore the need to explore alternative markers or characteristics, such as cell phenotypes and functional status, to achieve precise classification and holistic understanding of cell subpopulations. Additionally, while the article’s unsupervised clustering of HSCs based on WNN space reveals stable cell state heterogeneity among young HSC subpopulations, this finding overlaps with previous reports [5], necessitating further exploration. Furthermore, the distribution of mtDNA mutations across HSC subpopulations warrants scrutiny, particularly regarding the sensitivity of detection techniques.

In summary, this study leverages bioinformatics tools to transition somatic mutation data from bulk sequencing to mtDNA barcode detection, elucidating cellular status and heterogeneity. However, reliance solely on mtDNA mutation barcodes may over or underestimate clone complexity. Future integration of single-cell genotyping with ReDeeM promises to identify clones with driver mutations, significantly enhancing our ability to discern genetic and non-genetic mechanisms underlying cancer metastasis, progression, and treatment resistance. This approach holds promise for disease diagnosis and the development of mitochondria-targeted gene therapy drugs.

## Data Availability

Not applicable.
